# Validation of the Arabic linguistic version of the Danish Prostatic Symptom Score for benign prostatic hyperplasia associated with lower urinary tract symptoms

**DOI:** 10.1080/2090598X.2021.1892291

**Published:** 2021-02-20

**Authors:** Abdelwahab Hashem, Mahmoud Laymon, Mohamed T. Taha, Magdy Elshabrawy, Mohamed M. Ghorab, Ahmed M. Elshal, Khaled Z. Sheir

**Affiliations:** a Urology Department, Urology and Nephrology Center, Mansoura, Egypt; b Urology Department, International Medical Center, Cairo, Egypt; c Urology Department, National Nephrology and Urology Institute, Cairo, Egypt; d Urology Department, Shebin Elkom Teaching Hospital, Menofyia, Egypt

**Keywords:** Prostate, Arabic version, DAN-PSS, reliability, validity

## Abstract

**Objective**: To validate an Arabic version of the Danish Prostatic Symptom Score (DAN-PSS), a self-administered quality-of-life questionnaire.

**Patients and methods**: The reliability of the Arabic DAN-PSS was assessed by determining the internal consistency (Cronbach’s α coefficient) and by assessing the test–retest reliability (Kappa [κ] test). Inter-domain associations were examined using Spearman’s correlation coefficient (*r*). The discrimination validity was evaluated using receiver operating characteristic (ROC) curves. The sensitivity to change of the questionnaire and its individual items was assessed before and after intervention using a paired *t*-test.

**Results**: In all, 106 men (55 patients with BPH and 51 without BPH symptoms) were included. A high level of internal consistency amongst the three domains of the answered Arabic DAN-PSS questionnaire was observed (Cronbach’s α > 0.70). Also, there was a good correlation between storage and voiding (*r* = 0.75; *P* < 0.001) and post-micturition symptoms domains (*r* = 0.51; *P* < 0.001). Voiding and post-micturition symptoms domains also had a good correlation (*r* = 0.51; *P* < 0.001). The agreement between the test and retest scores had a κ value of 0.83 (*P* < 0.001). The ROC curve had an area under the curve of 0.98. The sensitivity to change comparing patients with BPH who received medical or surgical intervention revealed Arabic DAN-PSS mean (SD) scores of 34.7 (17.7) and 17 (8.7) before and after the intervention, respectively (*P* < 0.001).

**Conclusion**: The Arabic DAN-PSS is a clear questionnaire, valid, reliable, and responsive that can be used for BPH associated with lower urinary tract symptoms assessment and follow-up in clinical practice and research in Arabic-speaking patients.

**Abbreviations**: AUC: area under the curve; BPH: benign prostatic hyperplasia; CI: confidence interval; DAN-PSS: Danish Prostatic Symptom Score; DRE: digital rectal examination; ICIQ-MLUTS: International Consultation on Incontinence Male LUTS Questionnaire; ICS: international continence society; IPSS: international Prostatic Symptom Score; IPSS-Arb: Arabic version of the IPSS; LUTS: lower urinary tract symptoms; PSA: prostatic specific antigen; PSS: prostatic symptom score; QoL: quality of life; ROC: receiver operating characteristic; UTI: urinary tract infection.

## Introduction

BPH associated with LUTS (BPH/LUTS) affects >20%  of men aged 30–79 years, reaching 80% of men by the age of 70 years, and causes bother and impairs quality of life (QoL) [1]. The ICS Subcommittee divided LUTS into three groups: storage, voiding, and post-micturition symptoms [2].

The European Association of Urology (EAU) guidelines for male LUTS/BPH [3] recommend using validated symptom score questionnaires. The IPSS lacks assessment of incontinence, post-micturition symptoms, and bother caused by each separate symptom. The International Consultation on Incontinence Male LUTS Questionnaire (ICIQ-MLUTS) and Danish Prostatic Symptom Score (DAN-PSS) assess incontinence, post-micturition symptoms, and measure the bother of each symptom [3–5].

Symptom scores are supportive in quantifying and identifying which symptoms are predominant. These validated symptom scores are sensitive to symptom changes and can be used to monitor treatment [3]. Arabic is one of the six official languages of the United Nations and is commonly spoken by >420 million speakers across North Africa, the Middle East, and other parts of the world [6].

We thought that a validated Arabic version of the DAN-PSS would help assess prostate-related symptoms and QoL to improve patient management as a supportive tool in national and international research trials. Thus, the present study aimed to develop an Arabic version of the DAN-PSS and to assess its validity and reliability.

## Patients and methods

This prospective study of the development of the Arabic DAN-PSS was followed by an assessment of the validity and reliability of this Arabic version between May 2019 and August 2020 at a tertiary teaching hospital after approval by the Institutional Review Board. Patients complaining of BPH/LUTS, seeking medical advice and who could read were invited to participate in our trial. Exclusion criteria were neurological bladder, diabetes mellitus, or acute UTI. BPH was diagnosed clinically based on history, including the validated Arabic version of the IPSS (IPSS-Arb) and physical examination, including a DRE, serum PSA levels, uroflowmetry, and pelvi-abdominal ultrasonography.

The English DAN-PSS was initially translated into Arabic. Translation to the cultural context and lifestyle related to the Arabic language was performed by a bilingual native Arabic speaker having experience of the language translation of clinical symptom questionnaires (one urologist and one independent native Arabic-speaking professional translator who had English as their first foreign language). This Arabic version was back-translated. Back-translation from Arabic into English was carried out (one urologist and one independent native English-speaking professional translator who had Arabic as their first foreign language).

Review of the translation and back-translation by a panel of native Arabic and English speakers, respectively, to identify any meaning errors and discrepancies. Any differences between the versions were resolved through a consensus meeting between the translator and the urologists [7]. This revised version (definitive version) was used for the study (Appendix A).

Pre-testing was performed using a sample of 20 patients, who represented a sample of potential respondents to the questionnaire, to assess its comprehensiveness. They reported no problems in completing and answering the questions clearly and simply. Those 20 patients were excluded from the final analysis.

Based on the Hansen *et al*. [4] and the Hajian-Tilaki study [8], for estimation of diagnostic accuracy and area under the curve (AUC = 0.90) accuracy index with a given moderate marginal error of 0.05 and 95% CI, the calculated total sample sizes for both groups of symptomatic patients and controls was 106 patients.

The reliability of the Arabic DAN-PSS was evaluated for internal consistency using Cronbach’s α for each domain. A Cronbach’s α value of ≥0.70 was considered acceptable for internal consistency [9]. Domain structures were examined by inter-domain associations using Spearman’s correlation coefficient (*r*). A good correlation was considered when *r* was >0.5. The Arabic DAN-PSS stability was assessed by a 2-week test–retest analysis using the weighted Kappa (κ) test. The discrimination validity was evaluated by comparing the scores of cases with those of controls using receiver operating characteristic (ROC) curves [10] and Mann–Whitney test. The criterion validity was assessed by comparing the correlation between the Arabic DAN-PSS and validated IPSS-Arb. The responsiveness or sensitivity to change of the Arabic DAN-PSS was assessed using a paired *t*-test to compare the scores difference before and after the BPH treatments. All statistical tests were done using the Statistical Package for the Social Sciences (SPSS®), version 21 (IBM Corp., Armonk, NY, USA); with a *P* < 0.05 considered statistically significant.

## Results

In all, 106 men (55 patients with BPH and 51 normal subjects) were included in the assessment of the validity of the Arabic DAN-PSS. The mean (SD) age was 61 (7.8) and 28 (6.1) years in BPH and normal controls, respectively. With regards to the level of education, 57 (53.8%) of the participants held a university degree, 31 (29.2%) had graduated from high school, and 18 (17.0%) had completed primary education. Regarding the BPH group, the mean IPSS was 9.1, with a corresponding 95% CI of 7.7–10.6. The mean (95% CI) post-void residual urine volume was 60 (20–100) mL. The mean (95% CI) serum PSA level was 4.8 (2.5–7.1) ng/mL.

In the BPH group, we found a high level of internal consistency amongst the three domains of the answered Arabic DAN-PSS questionnaire with Cronbach’s α > 0.70 (Table 1). Also, there was a good correlation between the storage and voiding (*r* = 0.75; *P* < 0.001) and post-micturition symptoms domains (*r* = 0.51; *P* < 0.001). The voiding and post-micturition symptoms domains also had a good correlation (*r* = 0.51; *P* < 0.001; Table 1). The internal consistency (Cronbach’s α) and its association by Spearman’s correlation coefficient for each question of the Arabic DAN-PSS in all study groups are also shown (Table 2). There was a good correlation between the Arabic DAN-PSS and the IPSS-Arb (*r* = 0.86; *P* < 0.001).

**Table 1.** Internal consistency (Cronbach’s α) and inter-domain association by Spearman’s correlation coefficient in the BPH group

Domains	Cronbach’s α	Storage *r* (*P*)	Voiding *r* (*P*)
Storage	0.93		
Voiding	0.74	0.75 (<0.001)	
Post-micturition	0.71	0.51 (<0.001)	0.54 (<0.001)

**Table 2.** Internal consistency (Cronbach’s α) and its association by Spearman’s correlation coefficient (*r*) for each question of the Arabic DAN-PSS in the study groups

	**Q1** *r* (*P*)	**Q2** *r* (*P*)	**Q3** *r* (*P*)	**Q4** *r* (*P*)	**Q5** *r* (*P*)	**Q6** *r* (*P*)	**Q7** *r* (*P*)	**Q8** *r* (*P*)	**Q9** *r* (*P*)	**Q10** *r* (*P*)	**Q11** *r* (*P*)	**Cronbach’s α**
**Q1**: Hesitancy												0.91
**Q2**: Weak stream	0.40 (<0.001)											0.90
**Q3**: Incomplete emptying	0.46 (<0.001)	0.55 (<0.001)										0.90
**Q4**: Straining	0.25 (0.003)	0.37 (<0.001)	0.39 (<0.001)									0.89
**Q5**: Daytime frequency	0.25 (0.003)	0.45 (<0.001)	0.35 (<0.001)	0.61 (<0.001)								0.90
**Q6**: Nocturia	0.34 (<0.001)	0.46 (<0.001)	0.43 (<0.001)	0.60 (<0.001)	0.64 (<0.001)							0.90
**Q7**: Urge	0.55 (<0.001)	0.47 (<0.001)	0.52 (<0.001)	0.33 (<0.001)	0.41 (<0.001)	0.36 (<0.001)						0.90
**Q8**: Urge incontinence	0.24 (0.006)	0.41 (<0.001)	0.31 (<0.001)	0.75 (<0.001)	0.62 (<0.001)	0.58 (<0.001)	0.54 (<0.001)					0.90
**Q9**: Dysuria	0.33 (<0.001)	0.48 (<0.001)	0.40 (<0.001)	0.49 (<0.001)	0.50 (<0.001)	0.57 (<0.001)	0.60 (<0.001)	0.60 (<0.001)				0.90
**Q10**: Post-micturition dribbling	0.16 (0.08)	0.37 (<0.001)	0.31 (<0.001)	0.53 (<0.001)	0.49 (<0.001)	0.52 (<0.001)	0.30 (<0.001)	0.42 (<0.001)	0.41 (<0.001)			0.90
**Q11**: Stress incontinence	0.26 (0.002)	0.44 (<0.001)	0.32 (<0.001)	0.57 (<0.001)	0.57 (<0.001)	0.51 (<0.001)	0.40 (<0.001)	0.66 (<0.001)	0.45 (<0.001)	0.41 (<0.001)		0.90
**Q12**: Overflow/seeping incontinence	0.15 (0.09)	0.26 (0.003)	0.34 (<0.001)	0.68 (<0.001)	0.46 (<0.001)	0.52 (<0.001)	0.23 (0.007)	0.54 (<0.001)	0.40 (<0.001)	0.47 (<0.001)	0.78 (<0.001)	0.90

Q: question, *P: P* value, *r*: Spearman’s correlation coefficient.

**Figure 1.**
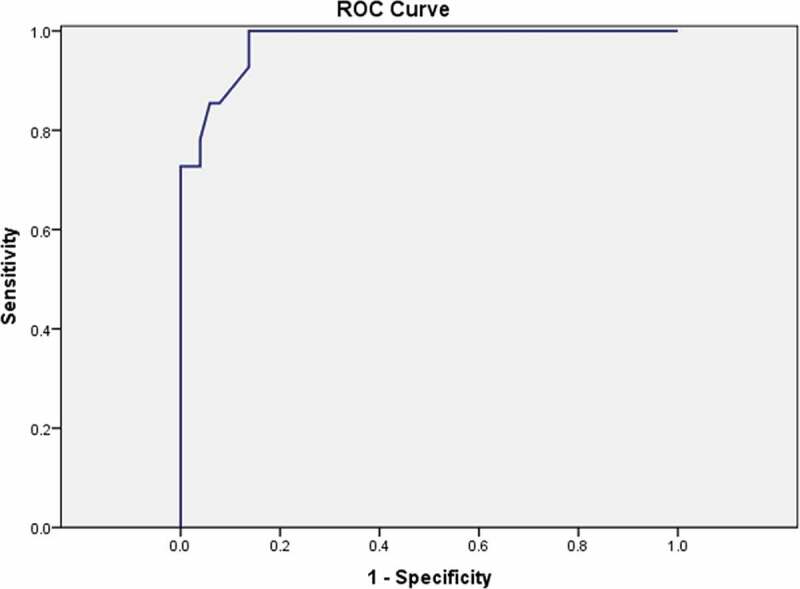
The ROC curve for the Arabic DAN-PSS. Total Arabic DAN-PSS ≥ 5 were the cut-off values associated with sensitivity of 98% and specificity of 86% for prediction of LUTS/BPH patients. The area under the curve (AUC) was 0.98 & p-value < 0.001

The agreement between the test and retest scores had a κ value of 0.83 (*P* < 0.001). The ROC curve constructed from the Arabic DAN-PSS total scores had an AUC of 0.98, which means that the Arabic DAN-PSS was able to discriminate between patients with BPH and control subjects who had no voiding problems in general 98% of the time. Using the ROC curve, a total Arabic DAN-PSS of ≥5 was the cut-off value associated with a sensitivity of 98% and specificity of 86% (*P* < 0.001) for prediction of patients with LUTS/BPH (Figure 1). There was also a significant difference between the median (range) Arabic DAN-PSS of patients with BPH 1 (0–11) and control subjects 19 (4–70) (*P* < 0.001). The sensitivity to change of the Arabic DAN-PSS was assessed in 26 patients with BPH who received medical or surgical intervention. The mean (SD) scores were 34.7 (17.7) and 17 (8.7) before and after the intervention, respectively (*P* < 0.001).

## Discussion

In men aged ≥40 years LUTS are highly prevalent and often bothersome symptoms, which have a major impact on health-related QoL, mental health, and workplace productivity that can result in economic effects [11,12]. Several patient-completed questionnaires have been developed to assess and follow-up men with LUTS including; the AUA symptom score, the IPSS, the DAN-PSS, and the ICIQ-MLUTS [4,13–15].

LUTS are divided into three groups: storage, voiding, and post-micturition symptoms [16]. As a result, the LUTS questionnaires differ in the range of LUTS symptoms that they can cover. The AUA/IPSS are short schedules [14,15], covering a few LUTS symptoms. On the other hand, the DAN-PSS and ICIQ-MLUTS questionnaires are longer and cover a much wider range of LUTS [4,13], including those associated with incontinence as well as measuring the bother of each symptom.

The DAN-PSS developed by Hansen *et al*. [4] includes the assessment of 12 LUTS: hesitancy, weak stream, incomplete emptying, straining, daytime frequency, nocturia, urge, urge urinary incontinence, dysuria, post-micturition dribbling, stress urinary incontinence, and overflow/sleeping incontinence [4,12].

The reliability of the Arabic version of the DAN-PSS, which refers to its ability to measure reproducibly [9] was confirmed by the high internal consistency and good inter-domain correlations as shown in Table 1. Also, there was no difference between the test and retest scores of the Arabic DAN-PSS indicating good stability. Similar results were found in the Korean linguistic validation studies [17]. This can be attributed to the high discriminatory power and good psychometric properties of the original English DAN-PSS [4] and the multi-step approach in the translation and back-translation process previously discussed [7].

The discriminant validity of the Arabic DAN-PSS revealed an area under the ROC curve [10] equal 0.98 (Figure 1), indicating that Arabic DAN-PSS correctly classified a randomly selected patient with BPH and control subject from the studied group 98% of the time. The criterion validity showed a good correlation between Arabic DAN-PSS and IPSS-Arb [18]. The Arabic DAN-PSS revealed a high degree of responsiveness of functional scales to clinical change and the improvements were also similar to those reported previously [19,20], supporting the Arabic DAN-PSS validity.

The limitations of our present trial are that the DAN-PSS used the standard Arabic language and tested it in a single hospital. It will need to be tested in different Arabic countries and communities, as the dialects differ from one country to another. We had some selection bias by excluding illiterate patients whom would need someone else to read and answer the questionnaire for them. Also, there was an age difference between the control and BPH/LUTS groups.

## Conclusion

The Arabic DAN-PSS is a clear questionnaire, valid, reliable, and responsive that can be used for BPH/LUTS assessment and follow-up in clinical practice and research in Arabic-speaking patients.

## Appendix A

A copy of the Arabic questionnaire is available from the corresponding author on request.
